# Plant hormone cytokinin at the crossroads of stress priming and control of photosynthesis

**DOI:** 10.3389/fpls.2022.1103088

**Published:** 2023-01-18

**Authors:** Martin Hudeček, Vladimíra Nožková, Lucie Plíhalová, Ondřej Plíhal

**Affiliations:** ^1^ Laboratory of Growth Regulators, Faculty of Science of Palacký University and Institute of Experimental Botany of the Czech Academy of Sciences, Olomouc, Czechia; ^2^ Department of Chemical Biology, Faculty of Science, Palacký University, Olomouc, Czechia

**Keywords:** priming, cytokinin, photosynthesis, stomata, ROS, stress, chlorophyll fluorescence

## Abstract

To cope with biotic and abiotic stress conditions, land plants have evolved several levels of protection, including delicate defense mechanisms to respond to changes in the environment. The benefits of inducible defense responses can be further augmented by defense priming, which allows plants to respond to a mild stimulus faster and more robustly than plants in the naïve (non-primed) state. Priming provides a low-cost protection of agriculturally important plants in a relatively safe and effective manner. Many different organic and inorganic compounds have been successfully tested to induce resistance in plants. Among the plethora of commonly used physicochemical techniques, priming by plant growth regulators (phytohormones and their derivatives) appears to be a viable approach with a wide range of applications. While several classes of plant hormones have been exploited in agriculture with promising results, much less attention has been paid to cytokinin, a major plant hormone involved in many biological processes including the regulation of photosynthesis. Cytokinins have been long known to be involved in the regulation of chlorophyll metabolism, among other functions, and are responsible for delaying the onset of senescence. A comprehensive overview of the possible mechanisms of the cytokinin-primed defense or stress-related responses, especially those related to photosynthesis, should provide better insight into some of the less understood aspects of this important group of plant growth regulators.

## Introduction

The crisis of feeding the world’s rapidly growing population with annual growth of around 80 million (Roser et al., 2013) is compounded by a plethora of conflicting issues, such as limited water availability or insufficient allocation of agricultural land. Moreover, various biotic and abiotic stresses resulting from long-term exposure to high temperatures and osmotic stress significantly limit crop yields. Water stress and salinity are considered to be the biggest challenges of present-day agriculture (Abhinandan et al., 2018), leading to poor seed germination rates, poor seedling emergence and poor stand establishment, thereby significantly limiting global crop production (Reed et al., 2022). Sustainable agriculture under the pressure of climatic change is one of the priorities of developed countries across the globe. The crop productivity is expected to have increased by approximately 60-100% by 2050; achieving such a goal without damaging the agricultural soil is challenging (Lau et al., 2022). Environmental stresses may be prevented by optimizing plant growth conditions and applying plant growth regulators (Yakhin et al., 2016). Seed priming – a process that involves seed imbibition with small amount of water to allow distribution of the priming agent – is recognized as an innovative and affordable technology to counteract harmful effects of abiotic stress by enhancing plant defense responses (Arun et al., 2022).

Similar to immunization in animals and humans, where an infectious agent or vaccine helps the immune system develop immunity to a disease, plants can also be immunized. This type of “acquired physiological immunity” in plants is now referred to as priming and can generally be described as the ability to elicit a faster and/or stronger defense response after a prior exposure to a biotic or an abiotic stress condition (Bruce et al., 2007; Hilker and Schmülling, 2019). Both biotic and abiotic stressors can elicit an adaptive response of the plant immune system, including changes at the epigenetic level, transcriptional reprogramming, and changes in protein phosphorylation (Conrath, 2011; Mauch-Mani et al., 2017). In general, priming allows plants to remember past stress events and prepare them for future attacks (Tsuda et al., 2013; Bjornson et al., 2014; Choi et al., 2014; Campos et al., 2016; de Zelicourt et al., 2016; Ariga et al., 2017; Chen et al., 2021). Linked multilevel processes generate a stress imprint or stress memory of a given priming event so that when a plant encounters the same specific condition, it can reach a primed state of enhanced and faster responsiveness and effectively regulate the relevant defense signaling cascade(s) (Conrath et al., 2006). Some excellent reviews address the molecular mechanisms underlying stress memory and priming formation upon various stress conditions in different plant species (Conrath, 2011; Conrath et al., 2015).

While defense priming is typically associated with bacterial and fungal pathogens or other biotic stressors, an altered response to abiotic stressors (also called hardening) is no less important. Previous exposure to a mild abiotic stress including heat, cold, osmotic or water stress can also improve resistance of various environmental stresses (Thomashow, 2010; Amooaghaie and Tabatabaie, 2017; Fan et al., 2018; Lemmens et al., 2019). Priming with plant hormones (hormopriming) is another popular and effective approach how to improve overall plant development (Iqbal et al., 2006; Nawaz et al., 2013; Chipilski et al., 2021; Vedenicheva et al., 2022), with cytokinins (CKs) emerging as a potentially interesting new group of hormopriming regulators that have been repeatedly shown to influence several developmental processes, including photosynthesis and senescence (Sobol et al., 2014; Vylíčilová et al., 2016; Hönig et al., 2018; Kučerová et al., 2020). In this review, we focus on possible CK effects in stress priming, with particular emphasis on the maintenance of photosynthesis, which is likely the backbone of the adaptive response mediated by this plant hormone.

## Cytokinin priming to increase crops growth and yield

It has been recently reported that priming of wheat seeds with benzylaminopurine (BAP) and kinetin (KIN) applied by spraying the wheat plants during the grain filling stage resulted in up to 14% higher productivity, higher fresh and dry weight, and chlorophyll content index of flag leaves (Chipilski et al., 2021). In addition to the positive impact on photosynthesis, another way in which CKs can influence plant fitness and yield is their natural ability to suppress oxidative stress, which has been also repeatedly associated with defense priming in plants (Kerchev et al., 2020). The offspring of CK-treated plants during the grain filling stage showed a lower accumulation of stress markers in field conditions demonstrating the transgenerational effect of this stress imprint (Chipilski et al., 2021). The CK-primed enhanced protection against oxidative damage was demonstrated on 15-day-old seedlings of wheat (cv. Geya-1) and these results positively correlated with a 25% lower average accumulation of malondialdehyde and hydrogen peroxide in 5-day-old seedlings of such wheat. In addition, the exogenous CK application in field conditions enhances the wheat seed viability after a low-temperature storage, which is an essential feature for practical agriculture (Chipilski et al., 2021). Moreover, KIN improved tomato shoot lengths after seed priming (Nawaz et al., 2013). As outlined above, the CK treatment can reasonably influence seed germination. This may be correlated with increased activity of hydrolytic enzymes in developing seeds, which can neutralize the impact of seed aging (Nawaz et al., 2013).

CK priming becomes particularly important when dealing with the consequences of climate change, such as temperature changes, increased solar radiation, drought, or salinity. The priming of rye seeds (cv. Boguslavka) with zeatin resulted in significant changes of endogenous CK pools in both shoots and roots of 7-day-old seedlings (Vedenicheva et al., 2022). Also, rye plants grown from CK-primed seeds were more resistant to hyperthermia stress than untreated control (Vedenicheva et al., 2022). Two cultivars of hexaploid spring wheat (*Triticum aestivum* L.) seeds were pre-soaked in BAP and KIN solutions, and the primed and non-primed seeds of salt-intolerant MH-_97_, as well as salt-tolerant Inqlab-91, were compared. KIN was effective in increasing the germination rate in the salt-intolerant cultivar and early seedling growth when compared with hydropriming under salt stress. Furthermore, the KIN-primed seeds showed a consistent promoting effect in the field and improved growth and grain yield in both cultivars under salt stress (Iqbal et al., 2006). The authors explain the priming effect by a complex crosstalk regulatory mechanism involving levels of active CK, abscisic acid (ABA) and indoleacetic acid (IAA) in developing plant leaves. CKs applied on the seeds can enhance the future shoot regeneration efficiency, as it activates the dedication of the shoot progenitor at later stages and allows chromatin to maintain shoot identity genes (Fathy et al., 2022; Wu et al., 2022).

Many CK-based compounds have already found application in micropropagation techniques and protection of plants against various types of abiotic stress, some were also tested experimentally in in field trials (Koprna et al., 2016; Plíhalová et al., 2016). Regarding seed priming, it may be interesting to note that the application of some CK-based derivatives, such as 2-substituted-6-anilino-9-heterocyclylpurine derivatives, has already been patented for seed dressing/coating of various crops, mainly cereals (Zatloukal et al., 2015). Their application led to increase of yield and quality of the agricultural product in harmful conditions (Zatloukal et al., 2015). Purine based (CK) derivatives were applied on maize, winter wheat and rapeseed. Other CK derivatives derived from urea were applied to fight stress caused by drought, heat or cold stress and salinity (Nisler et al., 2015). Such urea-based derivatives were applicated on winter wheat *Triticum aestivum* cv Hereward and spring barley (*Hordeum vulgare*), malting variety Bojos or winter oilseed rape treated by picking with a 50 µM solution of urea-based CK derivatives (Nisler et al., 2015). Since even seed coating where the active substances remain on the surface of the seed can cause such an impact on the crop productivity as described above, soaking the seed in the priming agents (although more technically demanding) may produce earlier and more consistent responses. Thus, it can have a more significant impact on crop yield.

## Mechanisms of cytokinin-mediated effects on photosynthesis

The reduction in photosynthetic capacity is associated with the reduced growth in many plant species exposed to stressful environments, demonstrating a direct relation between the photosynthetic capacity and crop yield (Moradi and Ismail, 2007; Naeem et al., 2010; Wu et al., 2012; Wu et al., 2019). Among the various biochemical processes, photosynthesis is highly sensitive to any environmental stress, with the photosynthetic apparatus being one of the most stress-sensitive plant components. Thus, photosynthetic capacity determined through the gaseous exchange (von Caemmerer and Farquhar, 1981) and chlorophyll fluorescence (Roháček, 2002; Lazár, 2015) measurements provides an excellent way to analyze the effects of stress on plants. In addition, monitoring photosynthetic variables by non-invasive techniques can be used to assess whether priming effects have been achieved and to determine the potential fitness benefits of the process. Optimization of the photosynthesis through transcriptomic reprogramming of target components of photosynthetic protein complexes or specific regulation of chlorophyll-related and other metabolic processes could, on the other hand, provide us with a powerful tool to increase yield under certain stress conditions, resulting in an overall improvement of agricultural production.

Priming, due to an inherently relatively high stability of photosystems and their high recovery response, offers a role in reducing the severity of stress and can therefore promote a rapid and complete recovery of plant physiological functions. Although different priming methods undoubtedly improve photosynthetic parameters and overall photosynthetic efficiency of plants exposed to various environmental stresses (Vincent et al., 2020; Sorrentino et al., 2021; Aswathi et al., 2022; Johnson and Puthur, 2022), the control mechanisms that govern the photosynthetic protection are still poorly understood. Recently, a major role in both the direct and indirect regulation of the photosynthetic protection under stress conditions has been attributed to various plant hormones (Müller and Munné-Bosch, 2021), which have long been known as effective priming agents (Kauss and Jeblick, 1995; Mur et al., 1996; Ton and Mauch-Mani, 2004; Hirao et al., 2012; Wang et al., 2014; Ji et al., 2021). We observed that CKs and their derivatives have important protective effects on the photosynthetic apparatus, which is manifested by the upregulation of photosystem components PSII (and more rarely PSI), upregulation of Calvin cycle components (RuBisCO, glyceraldehyde-3-phosphate dehydrogenase, etc.) and maintenance of chlorophyll through the downregulation of chlorophyll catabolism (chlorophyll b reductase coded by *NOL*, *NYE1*, *NYE2*, etc.; Vylíčilová et al., 2016) and the upregulation of chlorophyll biosynthesis, presumably through upregulation of protochlorophyllide oxidoreductase (Kusnetsov et al., 1998). This leads to a significantly better plant fitness and a delayed onset of senescence.

CKs are non-volatile plant hormones that reside mostly within plant vascular tissues and are unable to provide defense responses in neighboring plants (Dervinis et al., 2010). However, this does not seem to lessen their potential as effective priming agents. Apart from the relatively decent knowledge about their mode of action under optimal developmental and growth conditions, their role in plant defense priming related especially to photosynthesis is still a mosaic of individual findings. CKs play a central role in the chloroplast development and function and in chlorophyll biosynthesis (Cortleven and Schmülling, 2015). They are known to regulate many genes associated with photosynthesis (Brenner and Schmülling, 2012) and protect the photosynthetic machinery and productivity of plants exposed to various stresses (Chernyad’ev, 2009). CKs appear to act in the protection of photosynthesis at both levels, light and dark photosynthetic reactions, including the control of gas exchange.

Regarding the control of gas exchange by CKs, they are often considered as an ABA antagonist. Generally, exogenous CKs can inhibit ABA-induced stomatal closure in diverse species (Tanaka et al., 2006). The increased CK concentration in the xylem sap promotes the opening of stomata and reduces sensitivity to ABA (Daszkowska-Golec and Szarejko, 2013). Wild-type tomato leaves treated with CK showed enhanced transpiration and increased numbers of stomata per leaf area than untreated leaves (Farber et al., 2016). Mohammadi et al. (2015) demonstrated that BAP foliar application can significantly increase stomatal conductance in wheat under drought stress. An increase in internal CO_2_ concentration and water use efficiency (WUE) or increase in CO_2_ assimilation rate, stomatal conductance and transpiration was recorded under salt stress in eggplant after seedlings’ exposition to BAP (Wu et al., 2012) or in *Panax ginseng* plants at a later growth stage after inserting the stem base into BAP solution (Li and Xu, 2014). The naturally occurring zeatin-type bases, ribosides and *O*-glucosides supplied to the leaf in xylem sap regulated transpiration *in planta* in oat (Badenoch-Jones et al., 1996). Wheat priming by seed pretreatment with *cis*-zeatin or *trans*-zeatin significantly increased stomatal conductance, photosynthetic efficiency, shoot biomass with grain yield upon salt and drought stress (Alharby et al., 2020). Applying synthetic cytokinin, KIN, to *Tradescantia albiflora* leaves induced stomatal opening (Pharmawati et al., 1998). Seed priming with KIN alleviated the adverse effect of salt stress on gas exchange characteristics of wheat leading to improving growth and grain yield (Iqbal and Ashraf, 2005). Similarly, foliar spray of KIN on salinized mulberry plants increased the net photosynthetic rate (*P_n_*), WUE, carboxylation efficiency and leaf yield (Das et al., 2002). KIN could act as an effective priming agent also upon waterlogging stress, as reduced levels of reactive oxygen species (ROS), better water status, osmotic adjustment and an increased *P_n_*, WUE, improved growth and biomass under waterlogging were detected in primed mungbean plants (Islam et al., 2022a). Foliar-applied KIN remarkably improved maize performance by modulating growth, gas exchange and water related parameters under drought stress (Islam et al., 2022b).

The mechanism of direct action of CK on guard cells may involve the induction of membrane hyperpolarization by stimulation of the electrogenic H^+^-pump (Pospíšilová, 2003). The internal cytosolic free calcium concentration may mediate interactions between CK and ABA (Hare et al., 1997). In Kentucky Bluegrass, BAP has been proposed to promote stomatal opening through its effect on ABA balance leading to improved photosynthetic recovery from drought (Hu et al., 2012). CKs may promote stomatal opening also by scavenging H_2_O_2_ in guard cells as demonstrated in *Vicia faba* plants (Song et al., 2006). In addition, a higher carboxylation efficiency (*P_n_/C_i_*) was observed in *Anthurium* plants sprayed with KIN along with its possible effects on gas exchange and antioxidant enzyme activities (de Moura et al., 2018). Farber et al. (2016) proposed that CKs can act indirectly in stomata movement – CK levels reduced during adaptation to water deficiency suppress growth and reduce stomatal density, both of which reduce transpiration, thereby increasing the tolerance to drought. Priming by exogenous application of BAP to nutrient solution upregulated the RuBisCO large subunit content in some leaves of wheat plants (Criado et al., 2009). An incubation of wheat leaves in BAP solution reduced the degradation of the large and small subunits of RuBisCO (Zavaleta-Mancera et al., 2007). Foliar spraying of rice by synthetic CK (*N*-2-(chloro-4-pyridyl)-*N*-phenyl urea) reversed the drought mediated suppression of RAF1 and the RuBisCO activase proteins, implied in the assembly and activation of RuBisCO complex (Gujjar et al., 2020). Thus, CKs may also enhance photosynthesis at the molecular level by modulating the abundance of proteins related to stomatal conductance, chlorophyll content, and RuBisCO activity (Gujjar et al., 2020).

CKs may also protect the primary photochemistry processes of photosynthesis. An improved photosynthetic performance at donor and acceptor sides of the photosystem II reaction centre (RCII) was detected in wheat plants sprayed with BAP, which simultaneously increased their endogenous zeatin levels. A higher effective and maximal quantum yields of PSII photochemistry in the light (ΦPSII) and dark (*F_v_/F_m_*) adapted state, better transfers of electrons beyond Q_A_, enhanced electron transport rate and lower relative variable fluorescence intensity at the J-step were detected in the BAP primed wheat (Yang et al., 2018). In drought stressed maize seedlings, exogenous BAP priming regulated transient rise of fluorescence and increased the electron donation capacity of PSII (Shao et al., 2010). Application of BAP to the nutrient solution alleviated the detrimental effect of salt stress on primary photochemistry of eggplant by increasing *F_v_/F_m_*, *F_v_´/F_m_´* (maximal quantum yield of PSII photochemistry in light adapted state), ΦPSII and increase of *q_p_* (parameter of photochemical quenching) at the expense of non-photochemical quenching (NPQ) leading to a lower dissipation of excitation energy in the PSII antennae (Wu et al., 2012).

The protection of primary photochemistry by CKs can be attributed to their positive effect on the maintenance of chlorophyll pigments and the integrity of chloroplast membranes. Priming with BAP prior to water stress resulted in increased content of xanthophyll cycle pigments and their degree of de-epoxidation in four drought-exposed plant species (Haisel et al., 2006). Exogenously applied BAP significantly reduced the senescence-induced decrease in Chl, car, xanthophyll content and in the Chl/car ratio which was reflected in a lower impairment of PSII function in barley segments (Janečková et al., 2019). Seed priming by BAP significantly increased the content of photosynthetic pigments (chl a+b), car and carbohydrates in a salt stressed soybean (Mangena, 2020). Other mechanisms by which CKs maintain PSII functionality could be to stabilize Chl-protein complexes, both Light-harvesting complex II (LHCII) and RCII, as demonstrated during dark-induced senescing Arabidopsis leaves floating in BAP solution (Oh et al., 2005). In line with this, several genes encoding the light-harvesting chlorophyll a/b-binding proteins and various proteins of PSII are regulated by CKs in Arabidopsis (Brenner and Schmülling, 2012; Vylíčilová et al., 2016). CKs may also protect the cell membranes and the photosynthetic machinery from oxidative damage. Exogenous BAP reduced levels of ROS and enhanced the activity of antioxidant enzymes (CAT, APX) in dark senescing wheat leaves (Zavaleta-Mancera et al., 2007). Exogenously applied BAP alleviated the harmful effects of salt stress by increasing photosynthetic efficiency and activity of antioxidant enzymes (SOD, POD, APX, CAT) and by reducing malonaldehyde contents and O_2_^.-^ production in eggplant (Wu et al., 2012). Foliar application of KIN to *Vigna radiata* plants regulated antioxidant enzyme activities and reduced the increase in total peroxide, leading to suppression of the adverse effect of salt stress (Chakrabarti and Mukherji, 2003).

In addition to naturally occurring CKs or the notorious synthetic derivatives, a new avenue opens up for the possibility of studying the priming of the photosynthetic apparatus using purposefully modified CK derivatives. Exogenous KIN and its synthetic derivatives significantly protected lipid membranes from the negative effects of accumulated ROS in detached wheat leaves in the dark (Mik et al., 2011). Synthetic purine based halogenated CK derivatives increased PSII photochemistry efficiency, chlorophyll and carotenoid levels, and abundance of some LHCII components during Arabidopsis leaf senescence (Vylíčilová et al., 2016). Aromatic CK arabinosides (BAPAs) presumably act as a new type of priming agents that promote the plant innate immunity (PAMP-like responses) and positively affect leaf longevity (Bryksová et al., 2020). BAPAs treatment in detached Arabidopsis leaves indicated its action as a priming agent for the PTI (PAMP-triggered immunity) response (Bryksová et al., 2020) and at the same time markedly reduced the high light-induced cell membrane damage, accumulation of lipid hydroperoxides as well as PSII photoinhibition (Kučerová et al., 2020).

## Cytokinin crosstalk with other phytohormones in priming

At first glance, the role of CKs in plant defense and priming may seem auxiliary compared to other plant hormones and volatile compounds. The traditional plant defense response related to the hormones salicylic acid (SA), jasmonic acid (JA) and ethylene (ET) are interlinked in a complex network regulating plant immunity (Conrath, 2006; Mishina and Zeier, 2007; Pieterse et al., 2009; Engelberth et al., 2011; Fragnière et al., 2011; Pieterse et al., 2014). These compounds and their volatile derivatives are well-documented as priming agents in many physiological processes (Farmer and Ryan, 1990; Engelberth et al., 2011; Song and Ryu, 2018; Avramova, 2019; Zhao et al., 2020; Singewar et al., 2021). In last decades, the participation of CKs in biotic (Choi et al., 2011; Cortleven et al., 2019) and abiotic (Barciszewski et al., 2000; Wani et al., 2016) stress responses has also been demonstrated; however, less is known about the molecular aspects of CKs in priming. Similarities in the defense mechanisms mediated by CKs and other hormones or Green Leaf Volatiles (GLVs) suggest that CKs play a more important role in the priming process than previously thought.

CKs are essential in orchestrating an immune response, most likely through the crosstalk mechanisms with other hormonal pathways. Importantly, CKs are significantly involved in plant-pathogen interactions through crosstalk with the SA pathway (reviewed in Choi et al., 2011). The CK transcription factor ARR2 confers resistance to *Pseudomonas syringae* in Arabidopsis *via* interaction with SA response factor TGA3 and binding to the *PR1* promoter. In addition to the direct activation of defense responses by ARR2, CK pretreatment led to a hyperactivation of *PR1* transcription after pathogen inoculation (Choi et al., 2010). It was also shown that high concentrations of CK primed defense responses in Arabidopsis seedlings against the *Hyaloperonospora arabidopsidis* (Argueso et al., 2012). CK treatment alone resulted in slightly increased SA-related defense pathway gene expression. However, following the pathogen inoculation, it was further potentiated. The CK-responsive gene expression was diminished in *eds16* mutant plants with impaired SA biosynthesis. Thus, this crosstalk mechanism may help plants fine-tune the defense responses against biotrophic pathogens and adjust the effects of CK on plant immunity (Argueso et al., 2012).

In addition to the well-documented crosstalk with the SA pathway, the connection of the CK pathway with the JA pathway has also been reported. CKs can prime an immune response against herbivory through a crosstalk with the JA pathway, resulting in reduced emission of GLVs (Schäfer et al., 2015). GLVs were among the first well-documented compounds to effectively prime a plant’s defense system (Engelberth et al., 2004). *In planta* exposure to GLV *cis*-3-hexenyl acetate caused a rapid accumulation of JA and linolenic acid (LNA) in poplar leaves after herbivory (Frost et al., 2008). In line with this, Dervinis et al. (2010) reported higher JA and LNA levels after the CK pretreatment following wounding, while the CK treatment alone did not alter the JA and LNA levels in unwounded leaves. In addition, wounding and CK priming resulted in reduced larval weight gain (Dervinis et al., 2010).

Elevated levels of JA were also measured in a recent study on a CK-mediated resistance against brown planthopper in rice (Zhang et al., 2022). Using the CRISPR-Cas9 genome-editing approach, the authors generated two independent rice knock-out lines of cytokinin oxidase/dehydrogenase 1 (*OsCKX1*). Consequently, the mortality dropped to 30% in the case of *OsCKX1* knock-out plants upon an infestation by brown planthopper, compared to the 90% wild-type mortality upon infestation. A simple CK treatment positively regulated JA biosynthetic and an expression of JA-responsive genes. In addition, the CK-mediated resistance against the brown planthopper infestation was diminished in JA-deficient mutant *og1* (Zhang et al., 2022). Although previous studies have shown an antagonistic relationship between these two hormones, in the case of biotic stress defense responses and priming, the roles of CK and JA may be rather synergistic (Zhang et al., 2022).

The mechanisms of a crosstalk between GLV and CK pathways are much less understood but offer a significant potential for further study. It is tempting to speculate that there should be a regulatory mechanism to maintain a balance in leaf mining processes during herbivory. The existence of “green islands” with elevated levels of zeatins, isopentenyl adenine and other CKs has been reported during leaf-mining by galling insects (Giron et al., 2007). Colonization of plants by endophagous organisms thus requires a hormonal crosstalk, with GLVs playing a possible role in the process due to their volatile nature, which allows them to prime defense responses in places where CKs cannot overcome the natural barriers given by vascular constraints. Interestingly, after the treatment of *Nicotiana attenuata* leaves with the oral secretion of *Manduca sexta*, the CK pathway suppressed GLV esters emission (Schäfer et al., 2015). While JA concentrations are positively correlated with the activity of the CK pathway (Dervinis et al., 2010; Schäfer et al., 2015), the release of GLV esters was negatively correlated, suggesting that CKs control the balance between these two oxylipin classes (Schäfer et al., 2015).

As outlined by Schäfer et al. (2015), the activation of the classic CK signaling pathway after a CK application does not exclude the possibility of an alternative CK pathway involved in plant defense and priming. Priming enables plants to retain a “stress memory” and the priming state can last for multiple generations (Holeski et al., 2012; Hilker and Schmülling, 2019). For this reason, CK priming may also trigger various epigenetic modifications. CK-induced phenotypic changes in canola, such as increased surface area of petals, jagged edges of petals, and altered vasculature of flowers, are carried forward to the next generation of non-primed plants (Zuñiga-Mayo et al., 2018). This phenomenon can be achieved through the alterations of DNA methylation status. *S*-Adenosyl-L-homocysteine hydrolase (SAHH) appears to play a major role in this process (Li et al., 2008). Exogenous CK application induced the expression of the three cytosine DNA methyltransferase genes, *MET1, CMT3*, and *DRM2*, suggesting an important role of CKs in promoting DNA methylations in Arabidopsis (Li et al., 2008). Interestingly, downregulation of *SAHH* caused DNA hypomethylation, increased levels of CKs, and resistance against various plant viruses in tobacco (Masuta et al., 1995).

In addition, our aromatic CK derivatives that have been synthesized and tested retain certain properties of CK responses, such as delayed senescence and defense against pathogens, while the classical CK signaling pathway is only negligibly affected (Bryksová et al., 2020; Kučerová et al., 2020); therefore, an alternative mode of action for CK priming *via* the classical MAPK signaling cascade in PAMP-triggered immunity (PTI) responses and underlying epigenetic modifications cannot be ruled out. Experiments with these aromatic CK derivatives confirmed the synergistic relationship between CK and JA in defense priming, as the levels of JA and its metabolites were significantly elevated (Bryksová et al., 2020). However, a negative correlation was found between the senescence activity of aromatic CK derivatives and ET production (Kučerová et al., 2020). An ET production is generally known to be stimulated by exogenous CK treatment (Cary et al., 1995; Zdarska et al., 2015). Together with the JA pathway, ET regulates the herbivory-induced responses (Onkokesung et al., 2010). Interestingly, no stimulation of ET production was observed in aromatic CK derivative-treated wheat and Arabidopsis leaves (Kučerová et al., 2020).

While the effects of CK priming under biotic stresses are relatively well documented, mechanistic studies on CK priming in relation to abiotic stresses are just beginning to emerge. Previous works are mainly devoted to CK’s role in priming against heat stress and drought (Cheikh and Jones, 1994; Kang et al., 2012; Sobol et al., 2014; Wu et al., 2016; Wu et al., 2017). Kang et al. (2012) found that sensor histidine kinase (AHK) mutant lines, mainly *ahk2* and *ahk3*, show significant tolerance to dehydration. CK crosstalk with ABA may be significantly involved in dehydration tolerance, as *ahk* mutants have an increased level of expression of ABA-upregulated genes (Kang et al., 2012). CK pretreatment with KIN was able to further increase the survival rate of *ahk* single mutant lines after subsequent dehydration stress (Kang et al., 2012). This phenomenon can be explained by the interplay of increased expression of ABA-upregulated genes and a wide range of CK effects (including priming) in plant defense may not be linked to the classical CK pathway, as previously discussed.

## Conclusion and perspectives

**Figure 1:**
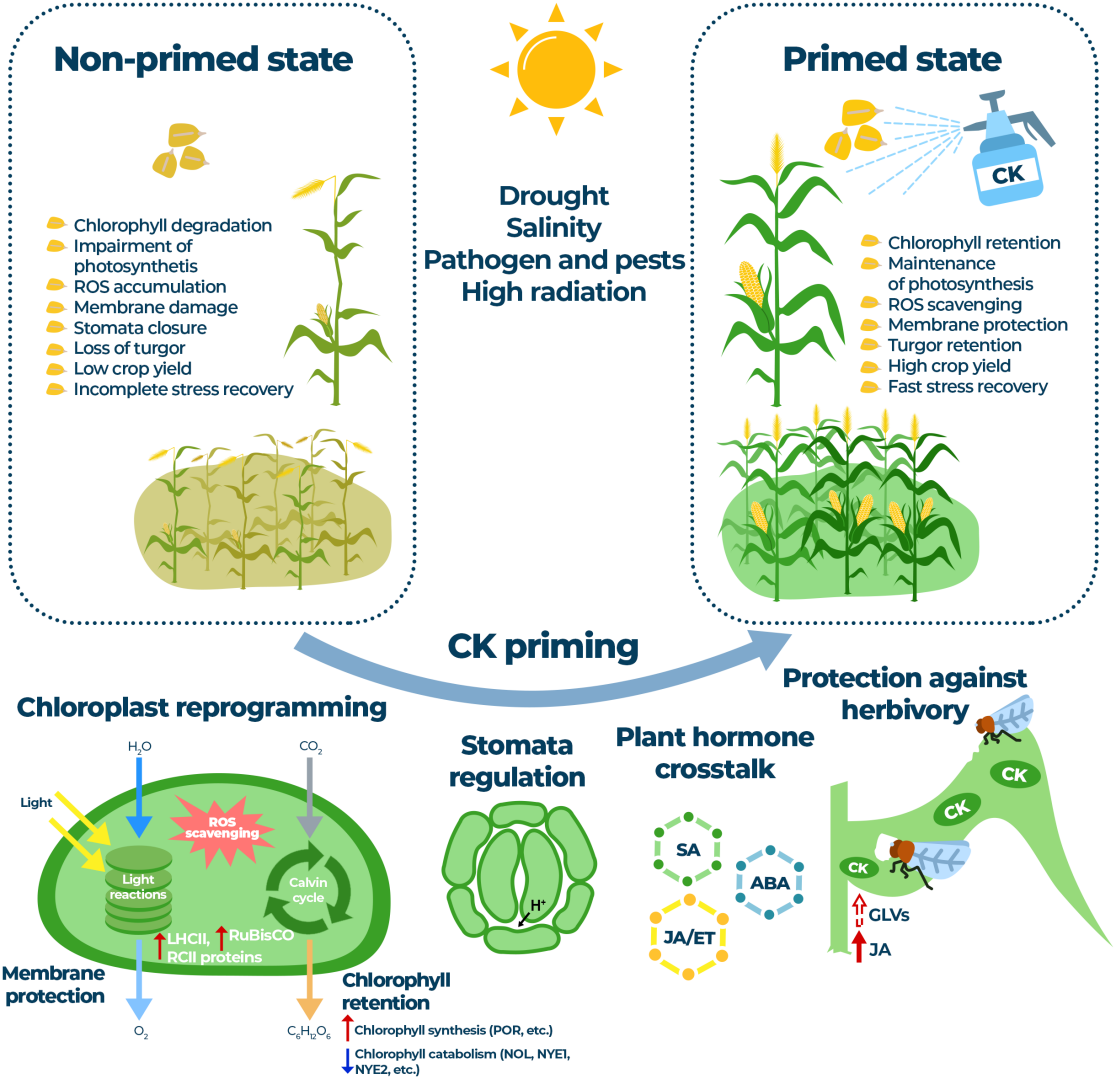
A simplified model showing the beneficial effects of cytokinin priming on photosynthesis and plant regulatory processes under stress conditions. Cytokinins can protect the light and dark reactions of photosynthesis by processes such as chlorophyll retention (upregulation of chlorophyll biosynthesis and downregulation of chlorophyll catabolism; cytokinin upregulated genes coding for *POR*, Protochlorophyllide oxidoreductase and downregulated genes coding for *NOL*, Chlorophyll b reductase; *NYE1*, Non-yellowing 1, and *NYE2*, Non-yellowing 2, are shown). Other cytokinin regulated processes include upregulation of LHCII and RCII components, upregulation of RuBisCO activity and activation of ROS scavenging system leading to membrane protection. Another important mechanism of action of cytokinins during stress and recovery is the desirable crosstalk of cytokinins with traditional plant stress hormones, potentially including volatile substances (GLVs). During herbivory, so-called “green islands” with elevated levels of CKs are formed; crosstalk mediated by volatile compounds and other phytohormones can initiate defense priming. Thus, the application of cytokinins can regulate plant response to various abiotic and biotic stresses, leading to balanced photosynthetic function, improved stress tolerance and increased crop yield.

In the last decade, many studies have pointed to the practical use of plant growth regulators that can mitigate the negative effects of biotic and abiotic stress conditions and improve overall crop condition and yield. Hormopriming is widely accepted as an efficient way to create memory imprints in seeds or young developing plants that can later, under limiting or stressful conditions, help shift the endogenous hormonal balance towards defense responses. Given the persistent social barriers to genetically modified crops, targeted modification of stress-related pathways through treatment with low amounts of highly bioactive substances offers an interesting alternative for agricultural use. The plant hormone CK is emerging as a promising new type of priming agents, the use of which appears to be closely linked to the control of the photosynthetic apparatus (**Figure 1**). Given the growing number of studies linking CK to defense or stress priming on the one hand and maintenance of photosynthesis on the other, more clear understanding on the mechanisms of a CK action on a photosynthetic and gas exchange machinery will be necessary for the more comprehensive understanding of CK-controlled stress protective responses. So far, mainly aromatic CKs BAP or KIN have been reported as priming agents with several possibilities of use. The application of natural CKs and many of their derivatives offers numerous advantages due to their negligible cytotoxicity to human cells and low risks to the environment resulting from their high biological activity in plants. However, although natural and some synthetic CKs show satisfactory results in terms of retarding senescence or maintaining balanced photosynthetic activity, they also show some negative effects on root development, and optimizing their effective use in field conditions remains a challenge. Therefore, new further SAR studies will be required to carefully promote the desirable traits of CK-based growth regulators to offer more practical future applications in sustainable agriculture.

## Author contributions

MH and VN wrote the original draft of the manuscript. VN designed the figure with help of OP. OP and VN conceived the idea and made the final adjustments. All authors contributed to the article and approved the submitted version.
